# Supplementary material for: Patterns of Intron Gain and Loss in Fungi
Source: PLoS Biol. 2004 Nov 30;2(12):e422. doi: 10.1371/journal.pbio.0020422 (PMC532390; doi:10.1371/journal.pbio.0020422)
Supplement: Table S1 — Also available at http://genes.mit.edu/NielsenEtAl/. (4.3 MB ZIP). [file pbio.0020422.st001.zip › NielsenEtAl/html/120.html]

AN0465.1.NCU08500.1.MG03251.1.FG00631.1


```
 CLUSTAL W (1.82) Multiple Sequence Alignments - Introns Inserted


Sequence 1: NCU08500.1	202 aa
Sequence 2: MG03251.1	202 aa
Sequence 3: FG00631.1	206 aa
Sequence 4: AN0465.1	201 aa
Alignment Length: 206 aa
Number Identitical Residues: 149 aa
Alignment Score (without introns) 6306


MG03251.1 	MGISRDSRHKRSATGAKRA~HYR1QKR~AWEAGRQPASTKIGAKRIHTVRVRGGNTKYRA
NCU08500.1	MGISRDSRHKRSHTGAKRA2YYR1KKR~AFEAGRQPANTRIGAKRIHTVRTRGGNHKYRA
FG00631.1 	MGISRDSRHKRSASGAKRA~YYR1KKR~AFEAGRQGANTRIGPKRVHTVRTRGGNHKYRA
AN0465.1  	MGISRDSRHKRSATGAKRA~HYR~KKR2AFEKGRQPANTRIGTKRIHLVRTRGGNRKFRA
          	************ :***** :** :** *:* *** *.*:**.**:* **.**** *:**

MG03251.1 	LRLDSGNFSWGSEGVTRKTRVIAVAY~HPSNNELVRTNTLTKSAVIQIDAAPFRQWYEAH
NCU08500.1	LRLDSGNFAWASEGCTRKTRVIVVAY1HPSNNELVRTNTLTKSAVVQIDAAPFRQWYEAH
FG00631.1 	LRLDSGNFAWGSEGLTRKTRVIAVAY~HPSNNELVRTNTLTKSAVVQVDAAPFRQWYEAH
AN0465.1  	LRLESGNFSWGSEGISRKTRVIVVAY~HPSNNELVRTNTLTKSAVVQIDAAPFRQWYEAH
          	***:****:*.*** :******.*** ******************:*:************

MG03251.1 	YGQSLGRRRQ----AKQAKEGEEVKKSKSVEKKQAERFAARGKVDSALEKQFEAGRVFAV
NCU08500.1	YGQPLGRRRQ----QKQGQVVEEVKKSKSVEKKQAERFAAAGKVDPALEKQFEAGRLYAV
FG00631.1 	YGQAIGRRRQKAQAAKEGKTEEEVKKSNAVEKKQAARMSAGGKVESAIEKQFEAGRLYAV
AN0465.1  	YGQPIGRRRQ----QKTAEATEE-KKSNSVVKKQAARFAESGKVESAIERQFESGRLYAV
          	***.:*****     * .:  ** ***::* **** *::  ***:.*:*:***:**::**

MG03251.1 	VSSRPGQSGRCDGYILEGEELAFYQRKLHK
NCU08500.1	ISSRPGQSGRCDGYILEGEELAFYQRKLHK
FG00631.1 	VSSRPGQSGRVDGYILEGEELAFYQRKLHK
AN0465.1  	IASRPGQSGRVDGYILEGEELAFYQRAIRK
          	::******** *************** ::*
```
